# Dual-energy CT material decomposition in the presence of nonuniqueness

**DOI:** 10.1088/1361-6560/ae28ae

**Published:** 2025-12-17

**Authors:** JP J Phillips, Emil Y Sidky, Ingrid S Reiser, Xiaochuan Pan

**Affiliations:** Department of Radiology, The University of Chicago, Chicago, IL 60637, United States of America

**Keywords:** nonunique, material decomposition, dual-energy CT, isoline

## Abstract

**Objective.:**

To develop a pathlength recovery method for material decomposition to address the challenge of nonunique mappings from fluence transmission to basis material pathlengths in dual-energy computed tomography (DECT) and implement it into a two-step algorithm to reconstruct material basis maps and virtual monochromatic images (VMIs).

**Approach.:**

By formulating the DECT imaging model as a system of implicit equations to form level curves, we present a projection-based decomposition method that explicitly accounts for cases of fluence transmissions mapping to multiple material pathlength combinations. Using numerical simulations, we investigate the method’s capability to recover and analyze multiple pathlength solutions, resulting in improved material basis map reconstruction and VMI synthesis.

**Main Results.:**

Examples of nonunique fluence mappings are demonstrated using polychromatic spectra (70 and 120 kV), demonstrating the potential for degraded material basis maps and VMI accuracy. We apply the pathlength recovery method to simulated DECT measurements of a digital phantom and demonstrate artifacts introduced by nonunique mappings if not properly resolved. A simulation with the VICTRE breast phantom is conducted to evaluate the quantitative accuracy of the method.

**Significance.:**

This work develops a method for resolving nonunique fluence transmission to material pathlength mappings in DECT material decomposition while furthering the theoretical understanding of DECT system behaviors. Our method address efforts to enhance reliability and accuracy of material decomposition that has implications relevant to general spectral CT imaging.

## 1. Introduction

Dual-energy computed tomography (DECT) exploits the dependence of x-ray attenuation on photon energy through the use of two spectral measurements when scanning an object to differentiate between materials. The idea of DECT is nearly as old as CT itself, with Hounsfield conceptualizing DECT in 1973 and the mathematical framework for processing DECT data being formulated by Hounsfield (1973), Alvarez and Macovski (1976). Early on, DECT showed improved ability to differentiate between different materials compared to conventional CT but suffered from inconsistent CT density values and long scan times that hindered widespread clinical implementation (Yoshizumi 2011, Goo and Goo 2017, Shayan et al 2020). Continued advancements in detector hardware, image reconstruction algorithms, and simultaneous spectral measurements has increased the clinical viability of DECT and led to applications in oncology, cardiology, and musculoskeletal imaging (Demehri et al 2023, Ramadhan et al 2023, Bolowia 2025).

Material decomposition is the foundation of numerous methods for DECT, yet there exist situations where material decomposition can be non-unique. In a 2017 theoretical study, Levine discovered the phenomenon of non-unique solutions in dual-energy reconstructions of two materials due to non-unique fluence mappings (Levine 2017). In this work Levine notes that the physical equations governing tomographic imaging, such as the Beer–Lambert Law, are nonlinear when considering multiple materials and beam hardening, indicating the reconstruction of two materials from two measurements may not be unique. He provided an example using basis materials of water and bone with spectra composed of three discrete photon energies. Later works investigated the uniqueness of fluence-to-pathlength mapping in relation to Jacobian determinant values and demonstrated examples of nonunique mappings for spectra comparable to those produced by clinical x-ray source tubes (Alvarez 2019, Phillips et al 2024). To date, there is no generalizable robust method for material decomposition capable of handling nonunique mappings. There have been no investigations into the effect of nonunique mappings on quantitative accuracy or what resulting artifacts would look like in clinical scans.

To address this challenge, we propose a projection-based material decomposition method that utilizes sinogram-to-transmission mapping level curves to recover material pathlengths. Material decomposition algorithms can broadly be separated into projection-domain, image-domain, and statistical frameworks. We choose to use a projection-based approach to directly analyze the physical forward model, offering a clearer insight into the existence and resolution of nonunique solutions. The method in this paper explicitly solves for two pathlength solutions if they exist, allowing for it to account for nonunique fluence mappings. Unlike conventional algorithms that consider that there will always be a unique mapping, our approach guarantees the recovery of the complete transmission fluence to material pathlength mapping for up to two pathlength solutions.

We investigate the theoretical basis of a novel basis pathlength recovery method, referred to as the isoline pathlength recovery method, for resolving nonunique mappings of fluence transmission to material pathlengths. We exemplify cases of nonunique fluence mappings using clinically realistic x-ray spectra and demonstrate the isoline pathlength recovery method’s ability to recover two valid pathlength solutions. We simulate DECT measurements for a digital phantom designed to generate nonunique fluence transmission values and apply the isoline pathlength recovery method in a two step material decomposition method to analyze the impact of nonuniqueness on reconstructed material basis maps and virtual monochromatic images (VMIs). The quantitative accuracy of the algorithm is validated by extending the technique for use in projection imaging material decomposition. Fluence measurements with two x-ray spectra are simulated using the VICTRE breast phantom for a two dimensional projection imaging (Badano et al 2018). Images are decomposed into water and iodine images followed by calculating the column concentration of iodine in each voxel. Our method provides a step towards furthering theoretical understanding of DECT imaging system properties and improving the accuracy of reconstructed DECT images.

## 2. Method

### 2.1. Data model

We consider a scanned object composed entirely of two materials. X-rays are generated from x-ray source tubes held at a low and high potential $V_{1}$ and $V_{2}$ respectively. Any x-ray beam that travels through the object along a ray will encounter some pathlength of each basis material $t_{1}$ and $t_{2}$ (both in cm). The x-ray beam will be attenuated depending on $t_{1}$ and $t_{2}$ and the energy dependent, nonnegative linear attenuation coefficient of each basis material $\mu_{1}(E)$ and $\mu_{2}(E)$.

The photon fluence, defined as the number of photons reaching the detector, from each x-ray source tube transmitted through the object along a single ray is given by:

(1) $$\phi_{i}\left(t_{1},t_{2}\right)=\int_{0}^{E_{max}}S_{i}\left(E\right)e^{-\mu_{1}\left(E\right)t_{1}-\mu_{2}\left(E\right)t_{2}}\;dE,i=1,2,$$

where $E_{\text{max}}$ is the maximum energy integrated over and $S_{1}(E)$ and $S_{2}(E)$ are the x-ray energy spectra corresponding to $V_{1}$ and $V_{2}$ respectively. X-ray spectra are assumed *a priori* and were modeled using the publicly available software toolkit SpekPy v2.0 (Poludniowski et al 2021).

In this work we use a standard linearization of the forward model by considering the negative log of the photon fluence referred to as the sinogram-to-transmission mapping:

(2) $$g_{i}\left(t_{1},t_{2}\right)=-ln\left(\phi_{i}\left(t_{1},t_{2}\right)\right),i=1,2.$$

Spectra are normalized such that $\int_{0}^{E_{\text{max}}}S_{i}(E)dE=1$. With the normalization, fluence values described by equation (1) will range from 1 to 0 for physical objects. Consequently, values of equation (2) range from 0 to infinitely large respectively. The goal of this work is to invert equation (2) to find $t_{1}$ and $t_{2}$ in terms of $g_{i}$.

We use a two step material decomposition method in order to reconstruct basis material density maps from measurements. In the first step, $t_{1}$ and $t_{2}$ are recovered from $g_{i}\left(t_{1},t_{2}\right)$ along each photon fluence ray to form pathlength sinograms for each material. Material density maps can be reconstructed from the sinograms using a reconstruction algorithm such as filtered-back projection (FBP).

The material decomposition process is straightforward when the mapping of fluence tranmission values to basis material pathlengths is unique. Previous work has shown that if the Jacobian determinant of the sinogram-to-transmission value is nonvanishing over the useful range of $t_{1}$ and $t_{2}$, then uniqueness is guaranteed (Bal and Terzioglu 2020, Terzioglu et al 2024). In this work, we are interested in developing an inversion method for the cases in which the Jacobian has a zero and the mapping is not necessarily unique.

### 2.2. Isoline pathlength recovery method

For a given transmitted fluence value $g^{M}$, there are several combinations of basis material pathlengths that can yield the same value. These pathlength combinations for the sinogram-to-transmission mapping in equation (2) can be denoted by a system of implicit equations as

(3) $$C_{i}\left(t_{1},t_{2}\right)=g_{i}\left(t_{1},t_{2}\right)-g_{i}^{M}=0,\;i=1,\;2.$$

**Figure 1.**
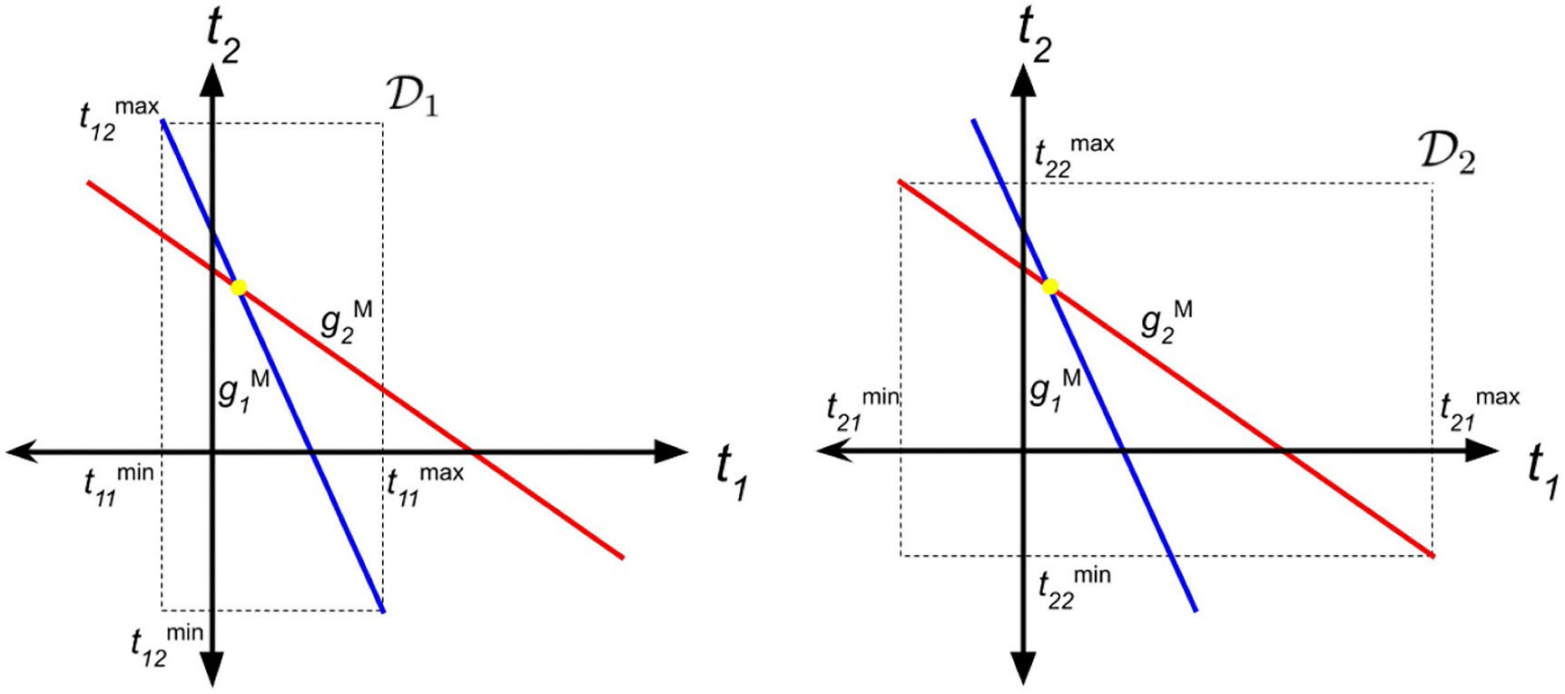
Example of level curves that satisfy $C_{1}\left(t_{1},t_{2}\right)$ and $C_{2}\left(t_{1},t_{2}\right)$ at the levels $g_{1}^{M}$ and $g_{2}^{M}$ respectively. The value of $g_{i}^{M}$ corresponds to a negative log fluence value for a source potential $V_{i}$ and basis materials $t_{1}$ and $t_{2}$. The intersection of the level curves (yellow point) indicates the values of $t_{1}$ and $t_{2}$ that satisfy equation (3). Domain bounds for $C_{1}\left(t_{1},t_{2}\right)$ (left) and $C_{2}\left(t_{1},t_{2}\right)$ (right) are marked.

The set of $t_{1}$ and $t_{2}$ values that satisfy each equation form the level curves of $g_{i}\left(t_{1},t_{2}\right)$ at levels $g_{i}^{M}$. Figure 1 shows an example of two level curves.

The level curves defined by the implicit equations in equation (3) exist in the space ($t_{1},t_{2}$). For each source spectra, level curve $C_{i}\left(t_{1},t_{2}\right)=0$ represents all pairs ($t_{1},t_{2}$) that would result in the measured fluence $g_{i}^{M}$. Physically, $t_{1}$ and $t_{2}$ should be nonnegative and their minimal values would equal zero. However, allowing negative values for $t_{1}$ and $t_{2}$ will preventing biasing solutions at the level curve bounding values for noisy measurements^1^. Each level curve's minimal values of $t_{1}$ and $t_{2}$, denoted as $t_{1i}^{min}$ and $t_{2i}^{min}$, respectively, can thus be selected freely. The maximal allowed value of $t_{1}$, denoted as $t_{1i}^{max}$, is defined as the value that satisfies $C_{i}\left(t_{1i}^{\text{max}},t_{2i}^{\text{min}}\right)=0$. Similarly, the maximal value of $t_{2}$, denoted as $t_{2i}^{\text{max}}$, satisfies $C_{i}\left(t_{1i}^{\text{min}},t_{2i}^{\text{max}}\right)=0$. The full domain for each $C_{i}$ is ${𝒟}_{i}=\left\{\left(t_{1},t_{2}\right)|t_{1i}^{\text{min}}⩽t_{1}⩽t_{1i}^{\text{max}},t_{2i}^{\text{min}}⩽t_{2}⩽t_{2i}^{\text{max}}\right\}$ and bounding values are marked in figure 1.

As a reminder, subscript i is used to denotes a quantity or function associated with the ith spectral measurement. When no subscript is shown, the notation refers to a single instance or a generic representative of that set. This convention will be important in the following discussion.

**Algorithm 1.** Pseudocode for expressing $\tau \left(t_{1}\right)$ at a given $t_{1}$ using n Newton’s method iterations with function $f\left(t_{2}\right)$ defined in equation (5). The value of n is key in efficiently and accurately finding $\tau \left(t_{1}\right)$. Floating point accuracy of 10^−8^ can reliably be found with at least n←10 when initialized at an approximate solution found by linear interpolation.

1:	**Input:** $g^{M},\;S(E)$, μ(E), $t_{1},\;t_{2}^{min},\;t_{2}^{max},\;n$
2:	$a\leftarrow f\left(t_{2}^{max}\right)t_{2}^{min}-f\left(t_{2}^{min}\right)t_{2}^{max}$
3:	$b\leftarrow f\left(t_{2}^{max}\right)-f\left(t_{2}^{min}\right)$
4:	$t_{2,1}\leftarrow a/b$
5:	**for** i←1 to n **do**
6:	$t_{2,i+1}=t_{2,i}-f\left(t_{2}\right)/\left(ℐ\left[\mu_{2},S;t_{1},t_{2,n}\right]/\phi \left(t_{1},t_{2,n}\right)\right)$
7:	$t_{2}^{*}\leftarrow t_{2,n}$
8:	**Return:** $t_{2}^{*}$

It is useful to reframe implicit equations in equation (3) as implicit functions of form $t_{2}=\tau_{i}\left(t_{1}\right)$.^2^ Using implicit functions allows for the two-dimensional equation (3) to be expressed as a function of one variable, $t_{1}$, which simplifies the mathematical processing while not removing the relationship of $t_{1}$ and $t_{2}$. Expressing function $\tau \left(t_{1}\right)$ explicitly is difficult as it would involve inverting equation (2). For any $t_{1}$, operationally $\tau \left(t_{1}\right)$ returns $t_{2}$ that will satisfy $g\left(t_{1},t_{2}\right)-g^{M}=0.\;\tau \left(t_{1}\right)$ represents the root of $g\left(t_{1},t_{2}\right)-g^{M}$ for a given $t_{1}$ and can be solved for using root-finding methods.

To calculate $\tau \left(t_{1}\right)$, consider a one-dimensional differentiable function f:R→R where Newton's method can be used to find the root x*∈R such that $f\left(x*\right)=0$ assuming the existence of a non-vanishing derivative $f^{\prime }\left(x_{n}\right)\neq 0$ in the area of the root. The iterative update step for Newton's method is given by

(4) $$x_{n+1}=x_{n}-\frac{f\left(x_{n}\right)}{f^{\prime }\left(x_{n}\right)}.$$

Given $t_{1}$, we let

(5) $$f\left(t_{2}\right)=g\left(t_{1},t_{2}\right)-g^{M}.$$

The Newton update step for a single level curve is thus

(6) $$t_{2,n+1}=t_{2,n}-\frac{f\left(t_{2,n}\right)}{ℐ\left[\mu_{2},S;t_{1},t_{2,n}\right]/\phi \left(t_{1},t_{2,n}\right)},$$

where

(7) $$ℐ\left[\mu_{j},S_{i};t_{1},t_{2}\right]=\int_{0}^{E_{max}}\mu_{j}\left(E\right)S_{i}\left(E\right)e^{-\mu_{1}\left(E\right)t_{1}-\mu_{2}\left(E\right)t_{2}}\;dE.$$

In equation (7) we define an operator style notation to simplify expressions which include multiple integrals such as equation (6). The expression in equation (6) is arrived at taking the derivative of equation (2) for the denominator.

The Newton update step is used calculate the implicit function $\tau \left(t_{1}\right)$ for a given $t_{1}$. To get in the area of the solution, a search interval $\left[t_{2}^{\text{min}},t_{2}^{\text{max}}\right]$ is defined and linear interpolation is used to approximate where $g=g^{M}$. Newton's method is then initialized at the approximation to achieve a more exact solution. This process will serve operationally as the implicit function $\tau \left(t_{1}\right)$ and is summarized in Algorithm 1.

In the two step material decomposition process, the first step is recovering the $t_{1}$ and $t_{2}$ responsible for the measured values $g_{i}^{M}$. These values correspond to where the level curves of $g_{i}\left(t_{1},t_{2}\right)$ intersect. Expressed using the implicit function $\tau \left(t_{1}\right)$, the intersection points will occur where the difference of the level curves is zero:

(8) $$d\left(t_{1}\right)=\tau_{1}\left(t_{1}\right)-\tau_{2}\left(t_{1}\right)=0.$$

The domain of $d\left(t_{1}\right)$, denoted as ${𝒟}_{d}$, will be the intersection the $C_{i}$ domains, $D_{1}\cap D_{2}$. Explicitly it is domain ${𝒟}_{d}=\left\{t_{1}|t_{1}^{min}⩽t_{1}⩽t_{1}^{max}\right\}$ where $t_{1}^{min}=max\left\{t_{11}^{min},t_{12}^{min}\right\}$ and $t_{1}^{max}=min\left\{t_{11}^{max},t_{12}^{max}\right\}$.

Solving equation (8) leads to the recovery of $t_{1}$ and $t_{2}$ mapping the fluence transmission values to basis material pathlengths. Consequently, multiple sets of $t_{1}$ and $t_{2}$ solving the equation would indicate non-unique mapping of the measured fluences. Based upon empirical observation, any pair of $\tau_{1}\left(t_{1}\right)$ and $\tau_{2}\left(t_{1}\right)$ will have, at most, two values of $t_{1}$ that satisfy equation (8). We will assume this to be true for the following work (see appendix A for discussion of when this assumption is not valid). We will also disregard the degenerate case where $\tau_{1}\left(t_{1}\right)=\tau_{2}\left(t_{1}\right)$ corresponding to using the same potential for both x-ray tubes.

**Figure 2.**
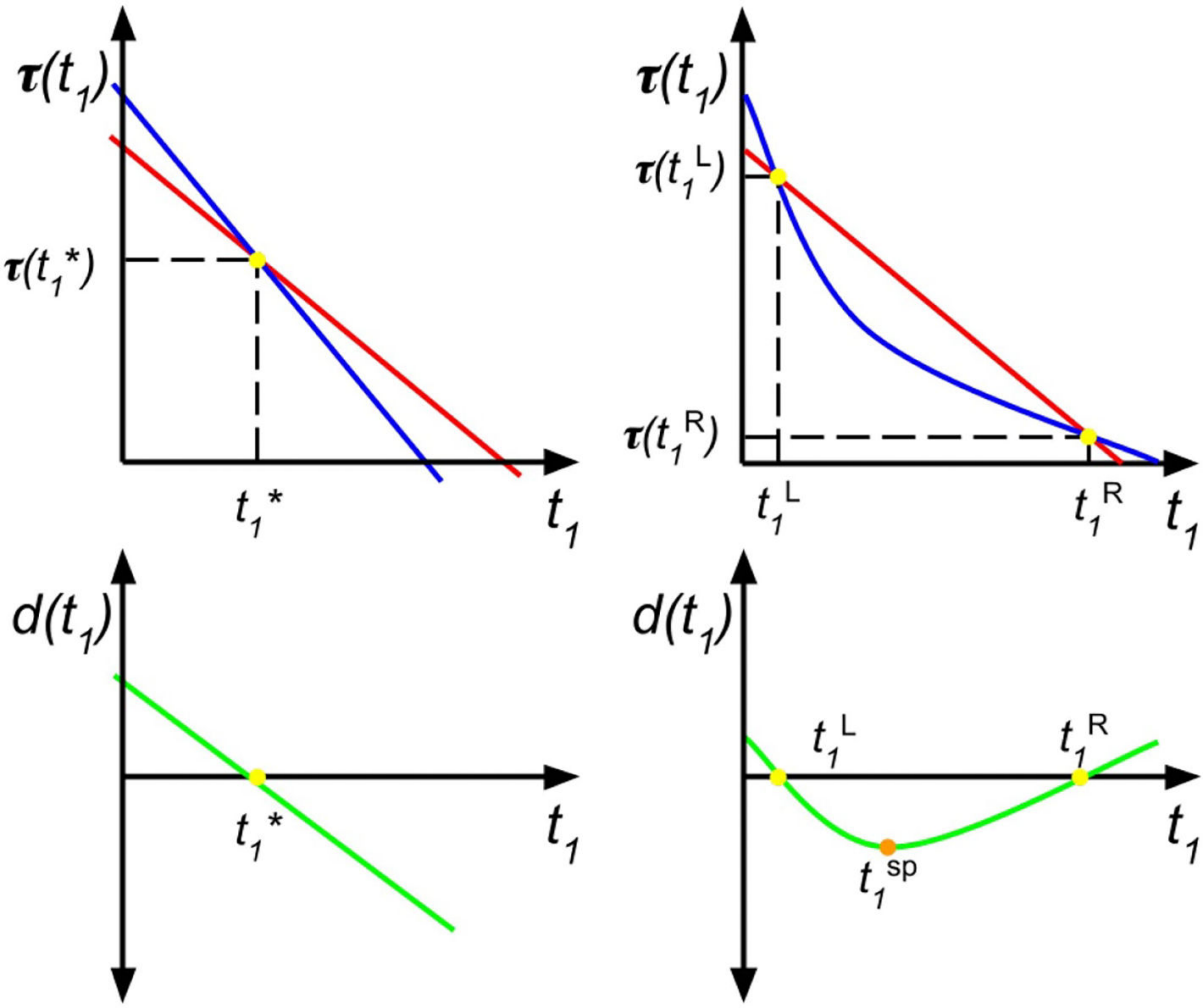
Diagrams of fluence level curves and difference curves. For one solution cases (left column), the value of $d\left(t_{1}\right)$ has difference signs at the boundaries of the domain, indicating only one solution exists. If two solutions exist (right column), then the sign of $d\left(t_{1}\right)$ is the same at the boundaries and a stationary point exists at $t_{1}^{sp}$ that has a different sign. One solution occurs to the left ($t_{1}=t_{1}^{L}$) and to the right ($t_{1}=t_{1}^{R}$) of $t_{1}^{sp}$.

The number of solutions can be established by checking the value of $d\left(t_{1}\right)$ at the bounding values of the $t_{1}$ domain, $t_{1}^{min}⩽t_{1}⩽t_{1}^{max}$. If $d\left(t_{1}^{min}\right)$ and $d\left(t_{1}^{max}\right)$ have different signs, then there is only one solution at $\left(t_{1}^{*},\tau_{1}\left(t_{1}^{*}\right)\right)$. If the signs are the same, then there are either zero or two solutions. Cases of zero or two solutions can be differentiated by finding the stationary point of the difference curve $t_{1}^{sp}$ defined as the value which satisfies $d^{\prime }\left(t_{1}^{sp}\right)=0$. Two solution cases must have a stationary point occurs within domain ${𝒟}_{d}$ and can be distinguished from zero solution cases by comparing $d\left(t_{1}^{sp}\right)$ to either d(0) or $d\left(t_{1}^{max}\right)$. If the sign of $d\left(t_{1}^{sp}\right)$ is different than the value at the boundaries, then there must be two solutions, $\left(t_{1}^{L},\tau_{1}\left(t_{1}^{L}\right)\right)$ and $\left(t_{1}^{R},\tau_{1}\left(t_{1}^{R}\right)\right)$, while if the sign is the same there are zero solutions. Diagrams of the one and two solution cases are drawn in figure 2.

To obtain and use knowledge of the number of solutions, three tools are needed: a method for solving equation (8) when there is only one solution, a method for finding the stationary point $t_{1}^{sp}$, and a method for finding both solutions to equation (8) when two exist. The derivation of these tools and pseudocodes for repeated high level functions are contained in appendix B.

The full isoline pathlength recovery method is summarized in Algorithm 2. The inputs for the algorithm include the bounding values of the $d\left(t_{1}\right)$ domain. As discussed previously, using lower bound $t_{1}^{min}⩽0$ is nonphysical and nonnegative values should not be allowed. For noiseless measurements this bounding value is sufficient. However, in the presence of noise the recovered values will be biased when one of the true pathlengths is zero. Setting lower bound $t_{1}^{min}<0$ removes the bias despite the nonphysical nature of the domain. $t_{1}^{\text{min}}$ is set empirically and only needs to be low enough that the distribution of solutions near $t_{1}=0$ is fully captured.

When two solutions are recovered, there is a challenge in determining which one is physically accurate to the scanned object. For this work, solution selection will be handled by either always using the solution from interval $\left[0,t_{1}^{sp}\right]$ or always using the solution from interval $\left[t_{1}^{sp},t_{1}^{max}\right]$. When no solutions exist, an average of $\tau_{1}\left(t_{1}\right)$ and $\tau_{2}\left(t_{1}\right)$ to return some estimate of the true pathlengths.

**Algorithm 2.** Pseudocode for the isoline pathlength recovery algorithm. The inputs $g_{i}^{M},\;S_{i}(E)$, and μ(E) do not appear explicitly in the pseudocode, but are required in order to calculate $\tau \left(t_{1}\right)$ throughout. The number of iterations n and m are important to ensure accuracy of the roots while maintaining stability and efficiency. Generally n←10 and m←5 will be sufficient for this purpose.

1:	**Input:** $g_{i}^{M},\;S_{i}(E)$, μ(E), $t_{1}^{min},\;t_{1}^{max},\;n,\;m$
2:	**if** $d\left(t_{1}^{min}\right)\times d\left(t_{1}^{max}\right)<0$ **then**
3:	$t_{1}^{\prime }\leftarrow Bisection\left(t_{1}^{\text{min}},t_{1}^{\text{max}},d\left(t_{1}\right),n\right)$
4:	$t_{1}^{*}\leftarrow Newton\left(t_{1}^{\prime },d\left(t_{1}\right),m\right)$
5:	**Return:** $\left[t_{1}^{*},\tau_{1}\left(t_{1}^{*}\right)\right]$
6:	**if** $d\left(t_{1}^{min}\right)\times d\left(t_{1}^{max}\right)>0$ **then**
7:	$t_{1}^{\prime }\leftarrow Bisection\left(t_{1}^{min},t_{1}^{max},d^{\prime }\left(t_{1}\right),n\right)$
8:	$t_{1}^{\mathrm{sp}}\leftarrow Newton\left(t_{1}^{\prime },d^{\prime }\left(t_{1}\right),m\right)$
9:	**if** $d\left(t_{1}^{min}\right)\times d\left(t_{1}^{sp}\right)>0$ **then**
10:	**Return:** $\left[t_{1}^{sp},\frac{\tau_{1}\left(t_{1}^{sp}\right)+\tau_{2}\left(t_{1}^{sp}\right)}{2}\right]$
11:	**else**
12:	$t_{1}^{\prime }\leftarrow Bisection\left(t_{1}^{min},t_{1}^{sp},d\left(t_{1}\right),n\right)$
13:	$t_{1}^{L}\leftarrow Newton\left(t_{1}^{\prime },d\left(t_{1}\right),m\right)$
14:	$t_{1}^{\prime }\leftarrow Bisection\left(t_{1}^{sp},t_{1}^{\text{max}},d\left(t_{1}\right),n\right)$
15:	$t_{1}^{R}\leftarrow Newton\left(t_{1}^{\prime },d\left(t_{1}\right),m\right)$
16:	**Return:** $\left[\left[t_{1}^{L},\tau_{1}\left(t_{1}^{L}\right)\right],\left[t_{1}^{R},\tau_{1}\left(t_{1}^{R}\right)\right]\right]$

Using the isoline pathlength recovery method, basis material pathlengths along each ray in a set of DECT measurements are estimated. The recovered values are arranged into separate basis material sinograms $s_{1}$ and $s_{2}$ corresponding to basis materials $t_{1}$ and $t_{2}$ respectively. $s_{1}$ and $s_{2}$ can then be linearly combined into a monochromatic sinogram as follows:

(9) $$s_{mono}=\mu_{1}(E)s_{1}+\mu_{2}(E)s_{2}.$$

where, for a VMI formed at an energy E, $\mu_{i}(E)$ is the attenuation coefficient for material i at that energy. VMI are then reconstructed from $s_{\mathrm{mono}}$ using FBP. The use of monochromatic sinograms for reconstructing VMI is chosen to ensure similar noise properties to standard CT (Macovski et al 1983).

### 2.3. DECT simulation setup

**Figure 3.**
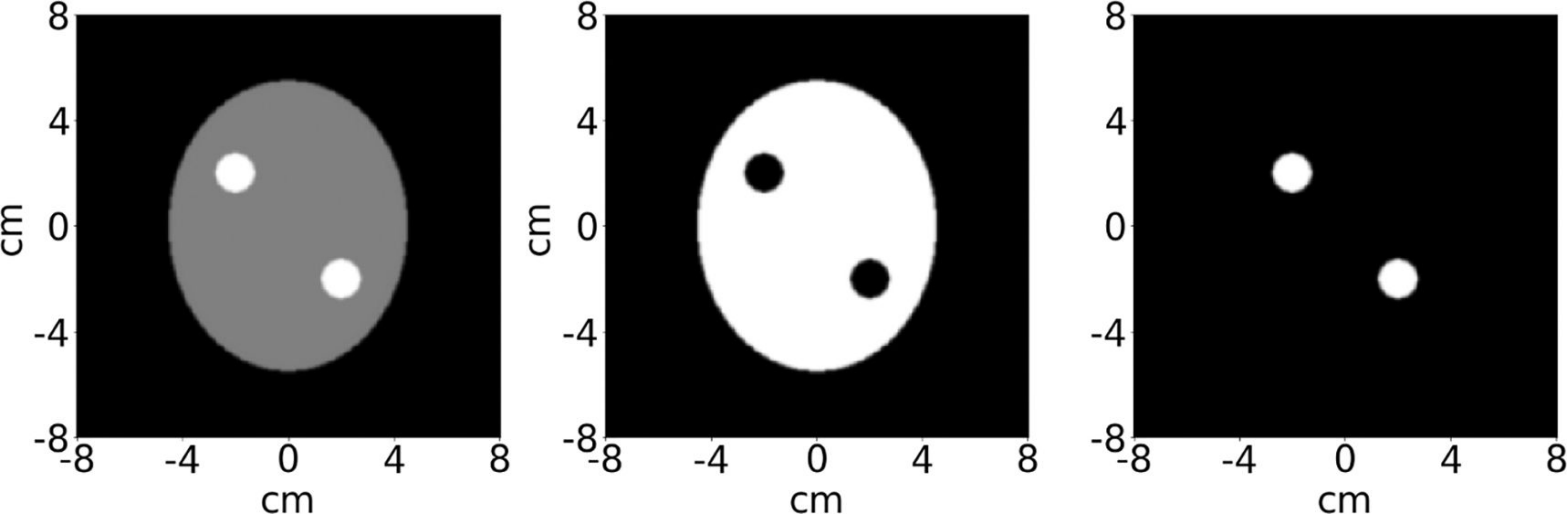
Plot of the elliptical water phantom with 79 mg ml^−1^ gadolinium solution inserts (left). The phantom consists of a ellipse-shaped water region (middle) and two circular gadolinium solution inserts (right).

For numerical simulations, a phantom consisting of an ellipse of water with two 79 mg ml^−1^ gadolinium solution inserts was used. The major and minor radii of the water ellipse was 5.48 cm and 4.50 cm respectively. The radius of the two gadolinium inserts was 0.73 cm. The ground truth phantom is shown in figure 3. DECT measurements of the phantom were simulated using two x-ray sources held at different tube potentials for 256 × 256 lines (bins × views) in fan beam geometry. The distance from the source to detector was 100 cm with a 50 cm rotation radius from the object isocenter to source. The simulation uses an ideal energy-integrating detector. Noisy measurements $g_{i}^{N}$ are simulated from noiseless measurements as $g_{i}^{N}=Pois\left(N^{0}g_{i}\right)/N^{0}$ where Pois(⋅) is the Poisson distribution function and $N^{0}$ is number of photons reaching each detector element (Whiting 2002). Simulations in this work use $N^{0}=10^{8}$ for both low and high kV scans. Aspects of the simulation, including phantom dimensions, contrast concentration, and spectra energy, where chosen to reflect a realistic scanning scenario while also allowing for nonunique fluence values to occur.

Pathlength sinograms for water and gadolinium were recovered from DECT measurements using the isoline pathlength recovery method and basis material maps were reconstructed using FBP. Monochromatic sinograms corresponding to 50 and 70 keV were formed and reconstructed into VMI using FBP. Additionally, 100 noisy realizations of DECT data were obtained to reconstruct average basis material maps and VMIs.

### 2.4. Projection imaging simulation setup

**Figure 4.**
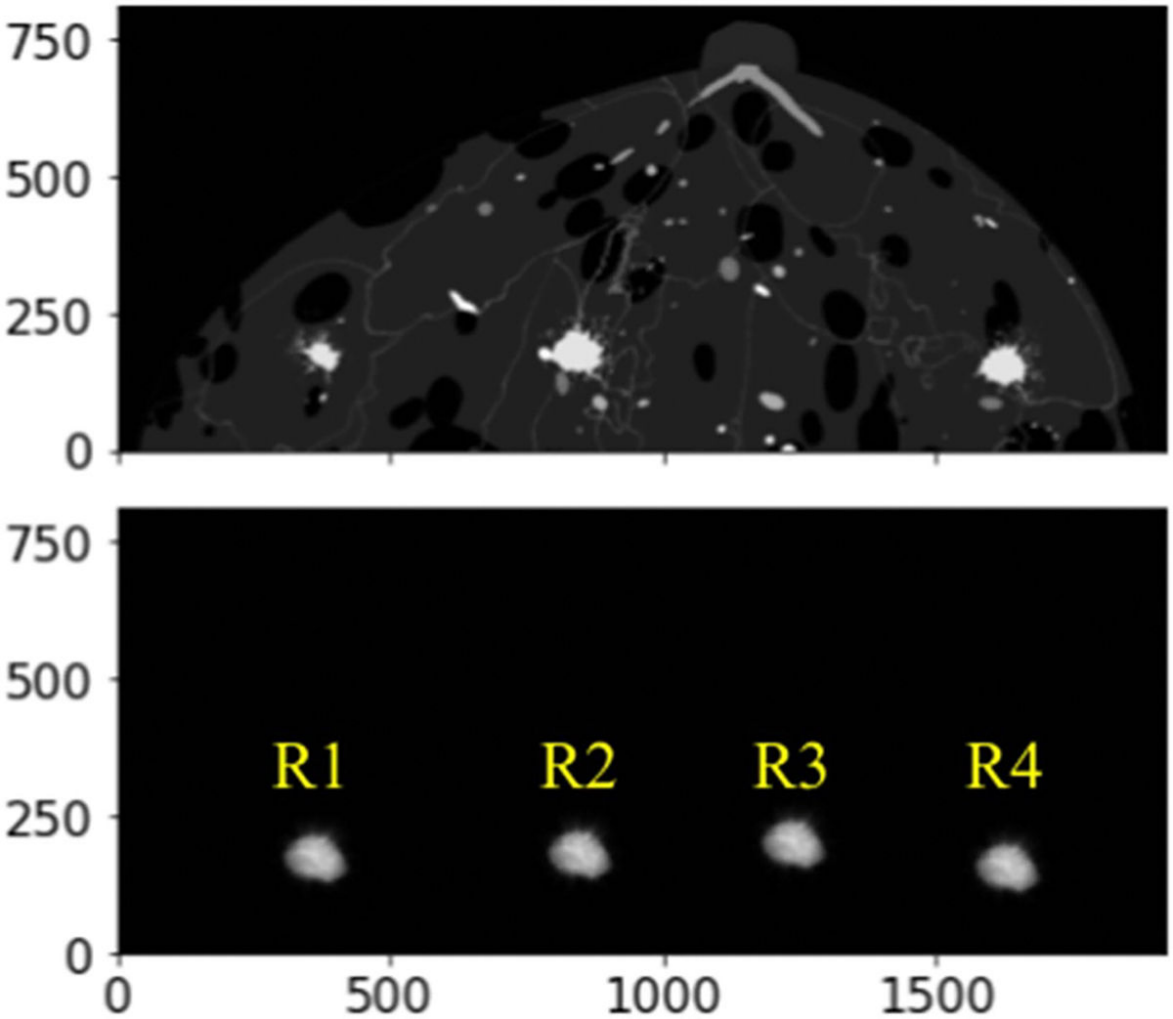
Single sagittal slice from labeled VICTRE breast phantom (top). The phantom includes four lesions where contrast is located. A map of the lesions flattened over the sagittal plane is shown to demonstrate the contrast distribution (bottom). The lesions are labeled R1 - R4 from left to right. Iodine solution concentrations from left to right are set to 10 mg ml^−1^, 30 mg ml^−1^, 50 mg ml^−1^, and 200 mg ml^−1^.

Quantitative accuracy of the isoline pathlength recovery method was assessed using a projection imaging simulation setup. To simulate a realistic anatomical composition, a sample dense breast phantom with lesions from the FDA VICTRE breast project was used (Badano et al 2018). The three-dimensional phantom is composed of 701 × 1920 × 810 voxels with dimensions 0.005 cm × 0.005 cm × 0.005 cm. The phantom contains four lesions which were replaced with iodine solution of varying concentrations (10 mg ml^−1^, 30 mg ml^−1^, 50 mg ml^−1^, and 200 mg ml^−1^). The solution concentrations were chosen to represent realistic concentrations for a breast projection image at varying values. The concentration of 200 mg ml^−1^ is much higher than what would be routine, but simulates the result if too much contrast solution is used. These parameters will allow for nonunique fluence measurements to occur. Figure 4 shows a slice of the phantom and the distribution of iodine through the sagittal plane. Parallel beam projection images were simulated through the sagittal plane using two x-ray sources to give 1920 × 810 images at low- and high-energies. Noisy measurements were simulated using $N^{0}=5\times 10^{4}$ for both low and high kV scans.

Material decomposition using water and pure iodine basis materials was completed by using the isoline pathlength method on the negative log fluence values at each pixel in the low- and high-energy images. With the recovered pathlengths of iodine in each pixel and the known dimensions of the phantom, the mass of iodine in each pixel can be quantified. To do this, first the volume of iodine ${𝒱}_{ℐ}$ in the pixel is calculated from the pixel cross-sectional area 𝒜 and pathlength of iodine $t_{ℐ}$:

(10) $${𝒱}_{ℐ}=𝒜\cdot t_{ℐ}.$$

The pixels have dimensions of 0.005cm × 0.005cm, meaning 𝒜 is 2.5 × 10^−5^ cm^−2^. Mass of iodine ${ℳ}_{ℐ}$ can then be found by multiplying the volume of iodine by density $\rho_{ℐ}$:

(11) $${ℳ}_{ℐ}={𝒱}_{ℐ}\cdot \rho_{ℐ},$$

where $\rho_{ℐ}$ is 4.93 gcm^−3^. While concentration of iodine itself cannot be recovered due to lack of three-dimensional data, cross-sectional concentration can be calculated by dividing ${ℳ}_{ℐ}$ by the pixel area 𝒜. For each pixel, the mass of iodine and cross-sectional concentration is calculated for the true iodine maps and recovered material maps.

## 3. Results

**Figure 5.**
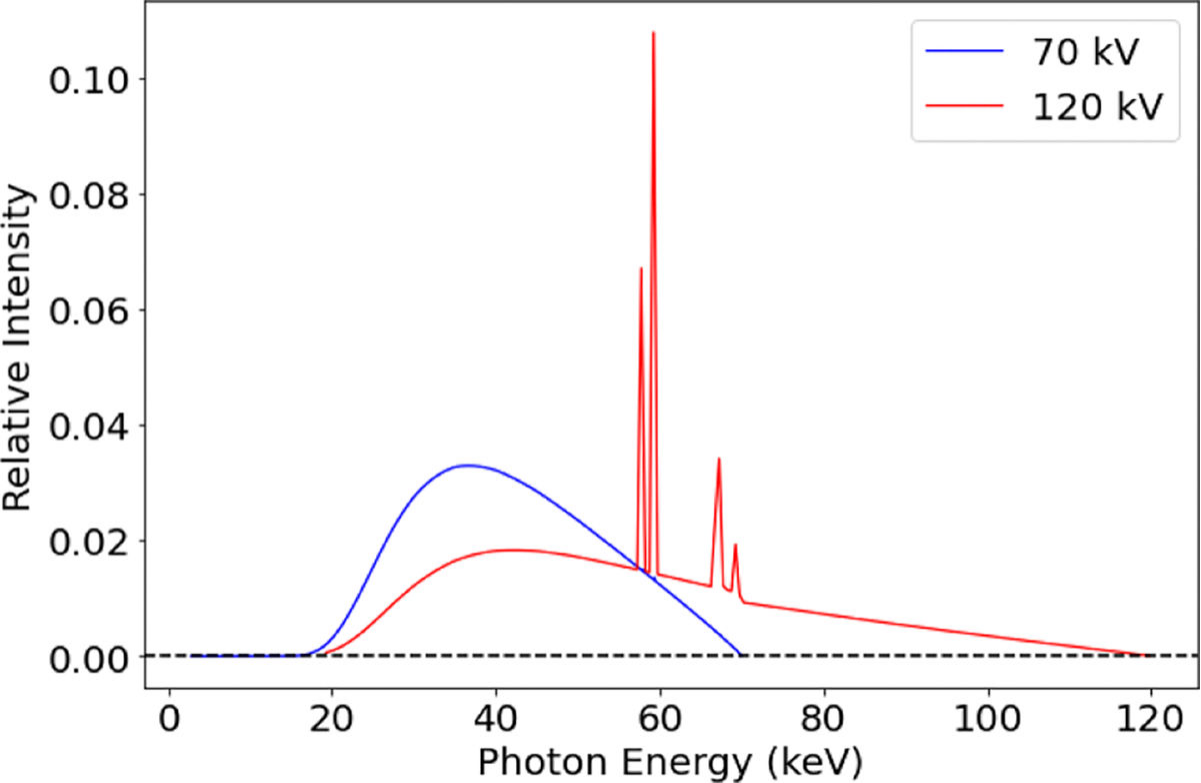
Spectra generated using SpekPy for x-ray source tubes held to at 70 and 120 kV. Both spectra used a tungsten target at a 12° angle with a 4 mm Al-filter.

As mentioned in section 2.2, fluence values that result in multiple solutions to equation (8) correspond to non-unique mappings to material pathlengths. To demonstrate the existence of nonunique pathlength values for realistic x-ray source spectra, consider the spectra generated from x-ray tubes held at a potential difference of 70 and 120 kV as shown in figure 5. The spectra are realistic to what a physical x-ray tube would generate and the tube voltages selected are within the routine range for clinical DECT scans (Johnson 2012, Forghani et al 2017). Using these spectra and material attenuation coefficients of basis materials looked up from the NIST database to simulate data for equation (2), a case where a pair of values for $g_{i}\left(t_{1},t_{2}\right)$ that can be generated from multiple values of $t_{1}$ and $t_{2}$ can be found (Hubbell and Seltzer 2004).

**Figure 6.**
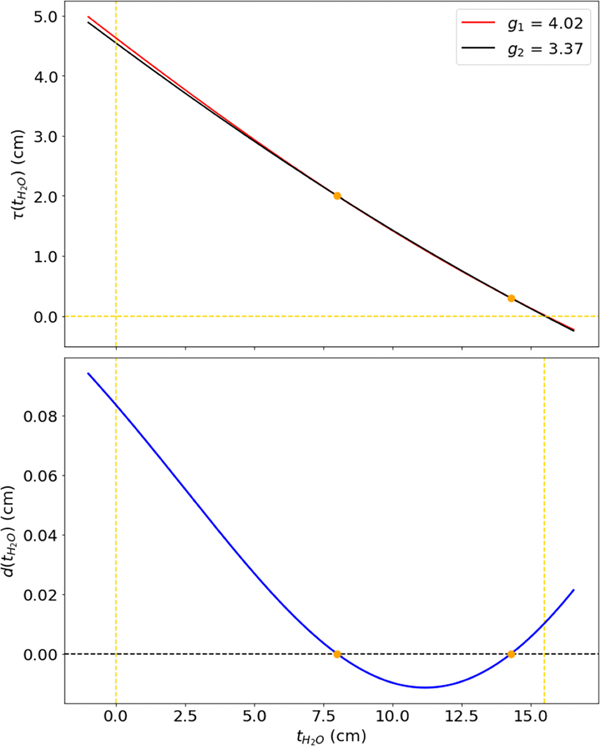
Isoline plots for negative log fluence measurements of water ($t_{1}=t_{H_{2}O}$) and gadolinium (79 mg ml^−1^)) solution $\left(t_{2}=t_{G}=\tau \left(t_{h_{2}O}\right)\right)$. Isolines are plotted in a space defined by the thickness of water and gadolinium solution (top). The red isoline corresponds to a 70 kV spectra measuring negative log fluence value of 4.02 and the black corresponds the 120 kV spectra measuring 3.37. Source spectra are shown in figure 5. The intersection of the isolines indicate solutions to equation (2) that are marked by the orange points. The vertical difference of the isolines (bottom) more clearly visualizes the two intersections. The left intersection occurs $t_{H_{2}O}=8.00\;cm$ and $t_{G}=2.00\;cm$ while the right intersection occurs at $t_{H_{2}O}=14.28\;cm$ and $t_{G}=0.30\;cm$. Boundaries where isoline cross into regions of negative pathlength are marked by yellow dashed lines.

Consider an object composed of water and gadolinium (79 mg ml^−1^) solution. Using the spectra from figure 5, isolines are plotted in figure 6 for $g_{1}\left(t_{1},t_{2}\right)=4.02$ and $g_{2}\left(t_{1},t_{2}\right)=3.37$ corresponding to the 70 and 120 kV sources respectively. The isolines in the figure intersect twice, indicating the existence of two solutions to equation (8) for the data. Thus, the fluence values do not have a unique mapping to water and gadolinium basis pathlengths.

To understand the effect of non-unique fluence mappings on basis map reconstruction, a numerical study was carried out for the phantom in figure 3. Noiseless data was generated using the 70 and 120 kV sources and the scanning configuration described in section 2.3. By applying the isoline pathlength method to the generated data, all material pathlengths were accurately recovered to computer precision using basis materials of water and gadolinium solution. The generated data included sets of fluence measurements which did not have a unique pathlength mapping. The isoline pathlength method recovered second solutions for these cases in addition to the true pathlength values.

Stability of the isoline pathlength method was assessed by generating 100 realizations of the same setup as the noiseless case with noise introduced using the method described in section 2.3. The two-step material decomposition method was subsequently applied, using the isoline pathlength recovery method to find the material pathlengths for each realization and averaging before reconstructing material basis images using FBP. Reconstructions for both always using the left or right solution for non-unique mappings were done.

**Figure 7.**
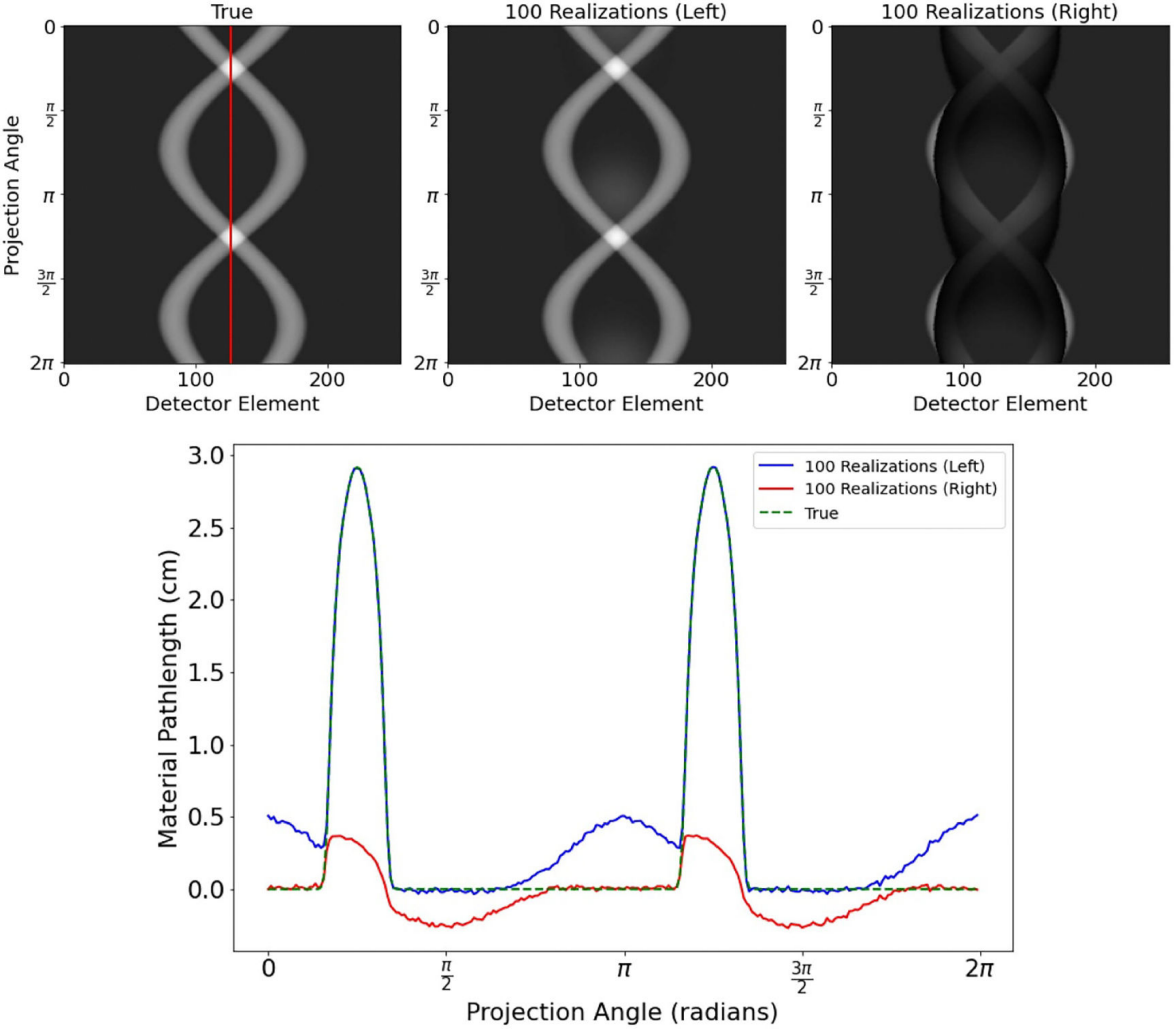
Gadolinium solution basis material sinograms for the simulated phantom. The true material pathlengths are shown (top left) and sinograms averaged over 100 realizations for always using the left solution (top middle) or right solution (top right). Window for the sinogram images is [−0.5, 3]. Pathlength values along the midline shown in the truth image were measured for each sinogram and are plotted (bottom).

Sinograms for gadolinium are shown in figure 7. The true sinogram is shown along with the mean sinograms resulting from 100 realizations always using either the left solution or right solution. Differences in sinogram values can be seen visually and numeric differences are demonstrated by the line profile plot of the vertical midline shown in figure 7. Instances of nonuniqueness where an incorrect gadolinium pathlength is recovered results in either underestimating or overestimating attenuation caused by gadolinium with a corresponding effect on the water pathlength recovered.

In the presence of noise, recovered material pathlengths are distributed around a mean pathlength approximately located at the true value. Even when multiple pathlength solutions are found, the average of one of the solution distributions will be located around the true value. Figure 7 demonstrates this result except in the regions π/2 to π and 3π/2 to 2π where neither of the averaged solutions match the true gadolinium pathlengths.

**Figure 8.**
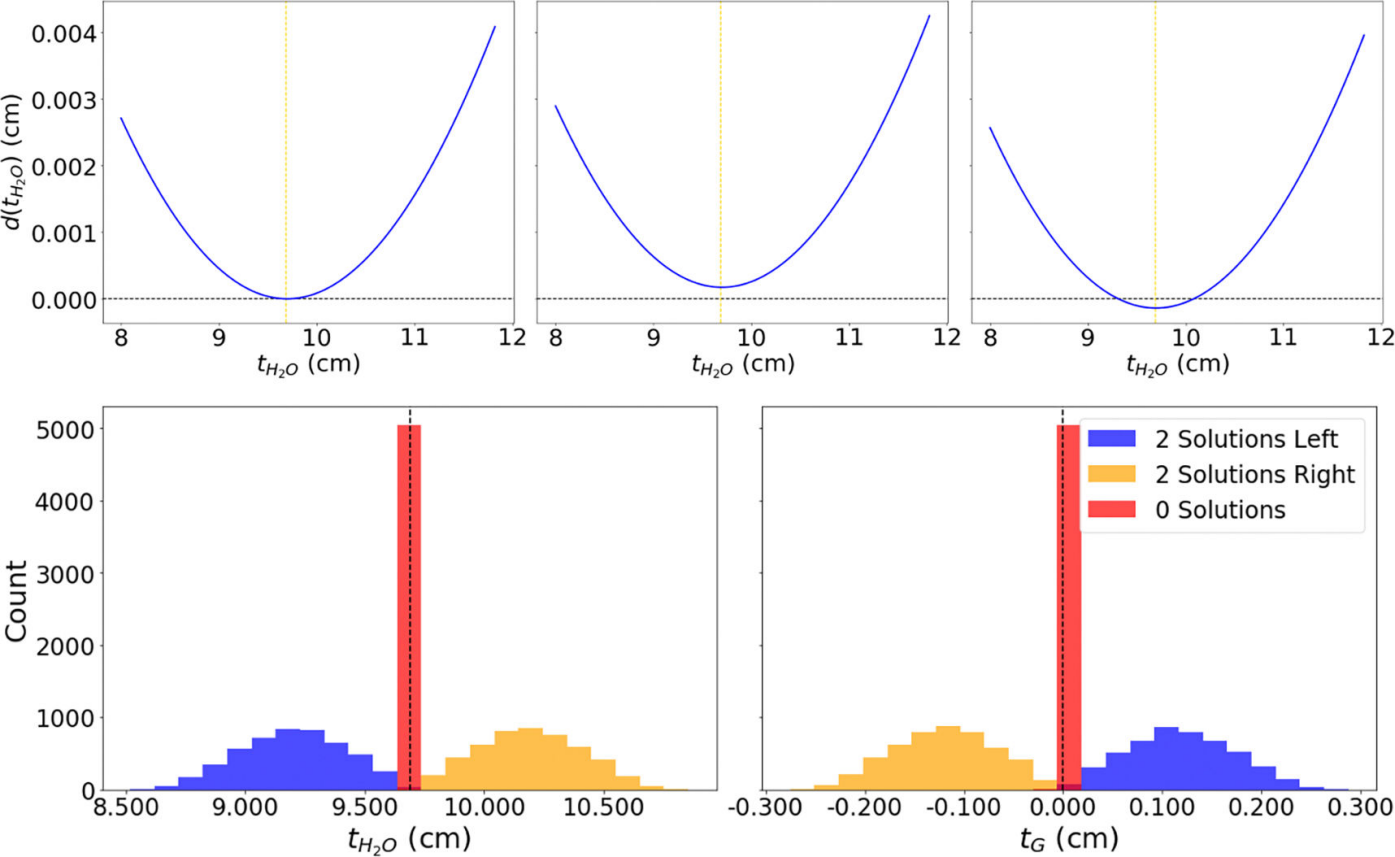
Example of how noise affects recovered pathlength solutions when difference curve roots are near a stationary point. Using 70 and 120 kV spectra, difference curves are shown (top row) for $t_{H_{2}O}=9.69\;cm$ and $t_{G}=0\;cm$. In the absence of noise (left), the roots of the curve are near its stationary point. Under noisy conditions the curve shifts to have either zero solutions (middle) or two solutions (right). Histograms of $t_{H_{2}O}$ (bottom left) and $t_{G}$ (bottom right) over 10 000 noisy realizations are shown with the true pathlengths marked by vertical dotted lines. The solution distribution for both materials has a bimodal Gaussian component corresponding to the left solutions (blue) and right solutions (yellow) for nonunique cases with a spike between the modes corresponding to the zero solution cases (red).

The cause of these regions can be understood by looking at noise realizations for a true material pathlength combination occurring in the biased region. Figure 8 shows effect of the difference curves shifting in response to noise for true pathlengths of 9.69 cm of water and 0 cm of gadolinium solution. The stationary point of the difference curves resides very close to the intersections points when there is no noise. As a consequence, the inclusion of noise will shift the difference curve up or down resulting in zero or two intersections respectively. Solving for the material pathlengths of 10000 noisy realizations and plotting them in a histogram in figure 8 reveals a bimodal distribution with a large spike between the two peaks. The two modes correspond to the two solution cases that roughly follow a Gaussian distribution and the spike corresponds to the zero solution cases. The result is that while the individual solutions in the two solution cases are normally distributed, the average of each is shifted by the large number of zero solution cases that causes the biased regions in figure 7. In such a case, a Gaussian noise distribution model is no longer appropriate unless the zero solution cases can be eliminated.

**Figure 9.**
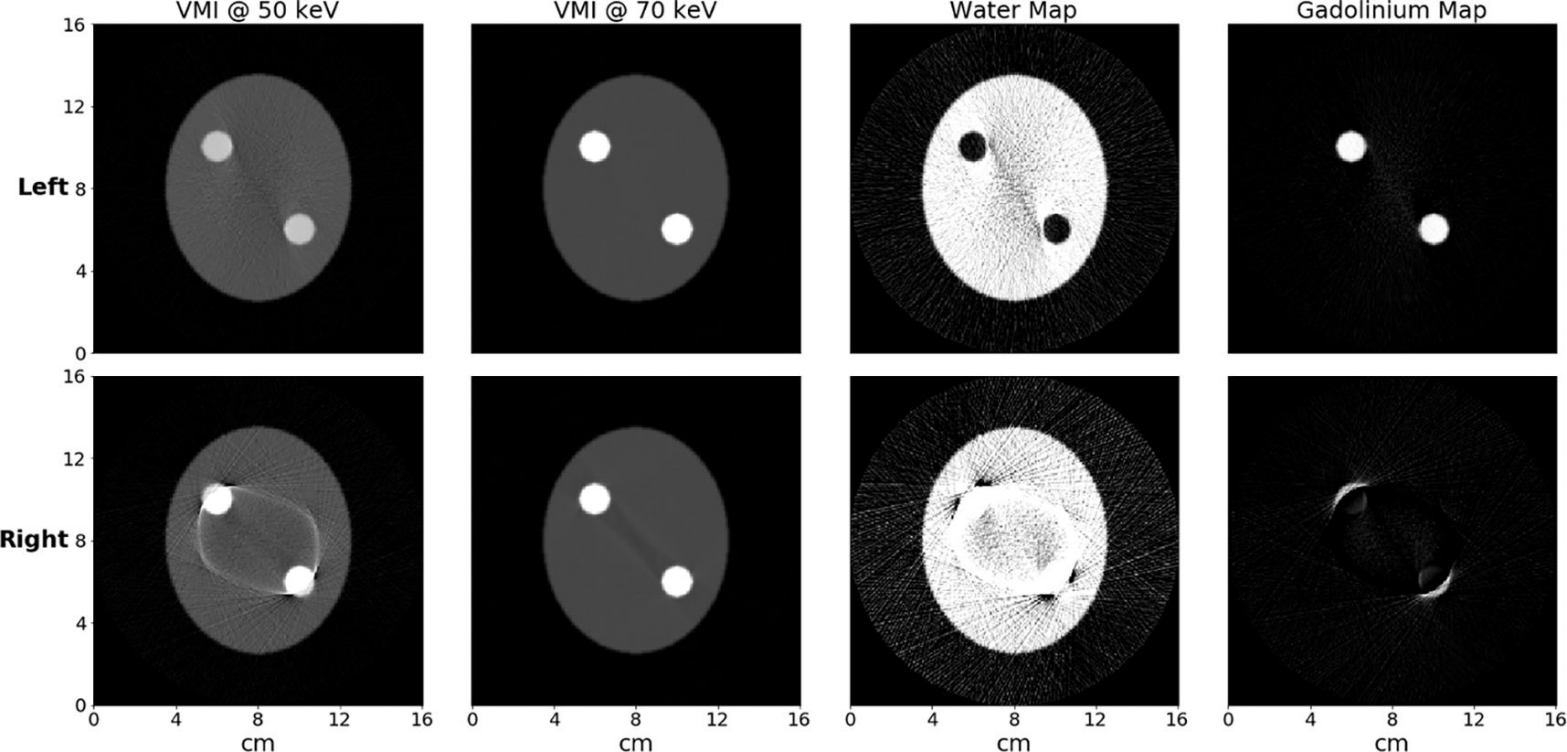
VMIs and material basis maps for water and gadolinium solution reconstructed from mean sinograms. VMIs were reconstructed at 50 keV (first column) and 70 keV (second column). Non-unique solutions were resolved by either using the solution to the left of the extrema (top row) or the solution to the right of the extrema (bottom row). Recovered water (third column) and gadolinium (fourth column) density maps are displayed for each solution selection method. Window for all images is [0,1].

Basis material images were reconstructed using FBP for both mean sinograms and are shown in figure 9. Inconsistencies with the ground-truth phantom can be observed in both material maps regardless of solution selection method. For this simulation, the left pathlength solution was more often the correct one as indicated by the less severe artifact in the images that used the left solution. Using equation (9), monochromatic sinograms are obtained by linearly combining the weighted basis sinograms. VMI are then obtained by applying the FBP algorithm to the monochromatic sinograms. VMI at 50 keV and 70 keV are displayed in figure 9. These energies are selected to show VMI formed above and below gadolinium’s 50.2 keV k-edge. Artifacts in the VMI are different based on both the selection method and VMI energy.

**Figure 10.**
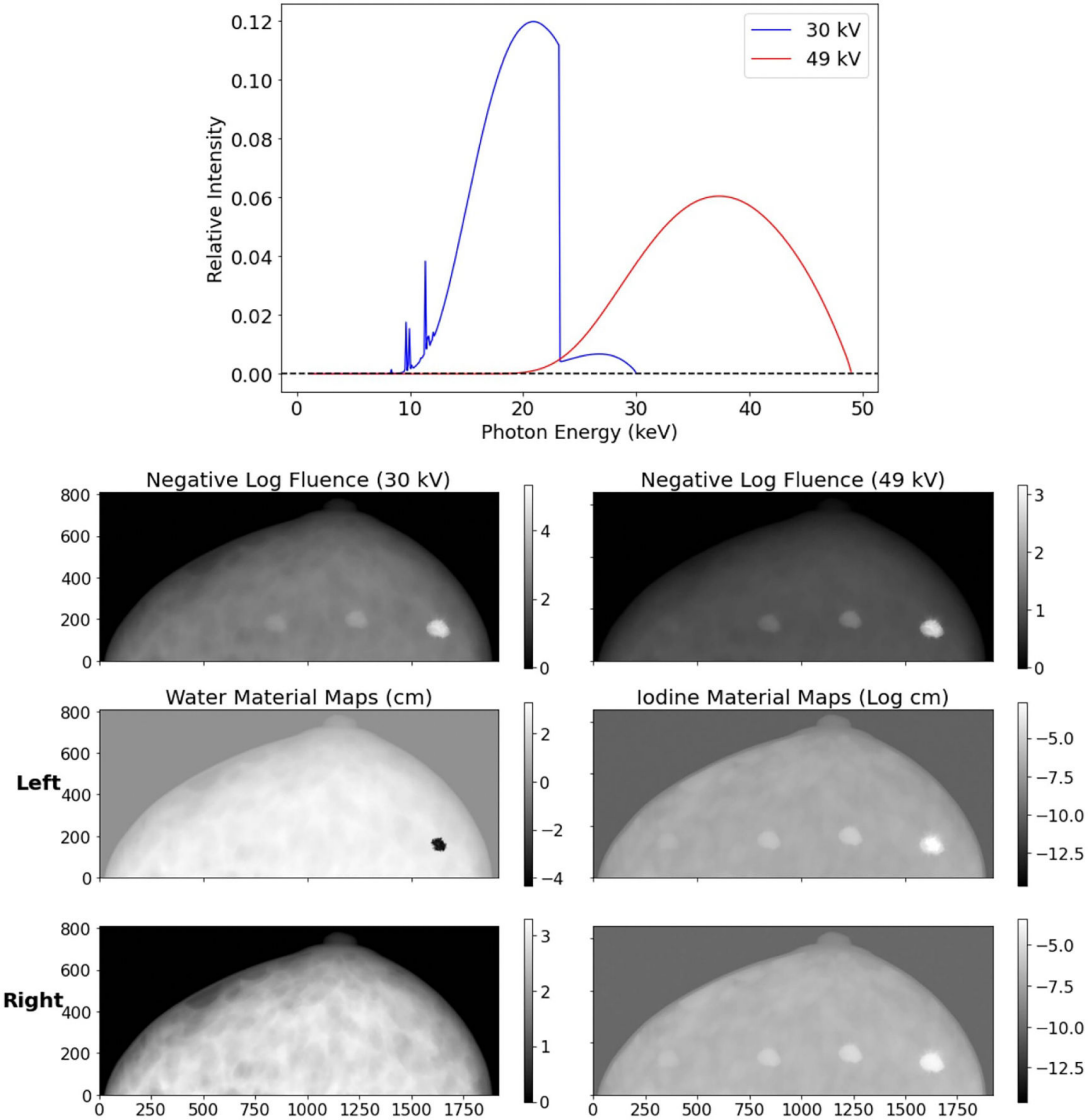
30 kV and 49 kV spectra used in projection image simulation (top row). Resulting negative log fluence projection images through the VICTRE phantom are shown (second row). Average water and iodine material maps from material decomposition over 100 realizations are shown either always using the left solution (third row) or right solution (last row).

For the single projection images of the VICTRE breast phantom, lower energy spectra are used to better differentiate between breast tissues. Spectra were generated from x-ray tubes held at potential differences of 30 and 49 kV. The 30 kV tube uses a 0.05 mm rhodium filter while the 49 kV tube uses a 1.0 mm titanium filter. Both tubes use a tungsten target. Spectra generated from these tube are shown in figure 10.

Results of simulating 100 noise realizations of projection images with the generated spectra and subsequent material decomposition using water and iodine basis materials are shown in figure 10. In the 200 mg ml^−1^ iodine area, nonunique fluence values occur and result in a difference in recovered material pathlengths when selecting either the left or right solution.

Iodine column concentration is quantified by calculating the average concentration in each contrast area. Water cannot fully represent that non-lesion areas of the phantom, leading to a low level iodine background being present over the entire object which would increase the measured column concentration. To account for the background, the average iodine pathlength at all non-contrast area pixels is calculated and subtracted from the contrast area pathlengths. Concentration values in each region R1, R2, R3, and R4 averaged over 100 noise realizations for the true map, left solution map, and right solution map are shown in table 1. The recovered iodine maps slightly over estimate the true column concentration values, indicating there is likely background iodine signal not accounted for. There is a large difference in recovered concentration for the 200 mg ml^−1^ region when using the left solution while the right solution is similar to the true value. The difference indicates the right solution is more likely the true pathlength of iodine and results in a more quantitatively accurate concentration measurement.

## 4. Discussion

In this work, we developed an isoline pathlength recovery method for two step projection-based material decomposition in the presence of nonunique mappings of fluence transmission to material pathlength. The developed method uses properties of imaging model level curves to determine if two fluence transmission values will map to zero, one, or two distinct material pathlength combinations.

We applied the isoline pathlength recovery method to simulated DECT measurements of a digital phantom containing water and gadolinium solution. The study demonstrated the effect nonunique fluence mappings have during material decomposition, resulting in erroneous material basis maps leading to novel artifacts in subsequently reconstructed VMI. The isoline method was also use for material decomposition of single projection images using the FDA VICTRE phantom with varying iodine solution concentrations. The study showed the effect nonuniqueness can have when quantifying iodine concentration from measurements and the possible importance of having a robust method for addressing nonunique fluence values. The study also demonstrates how nonuniqueness is not exclusive to DECT and can have consequences for other material decomposition modalities.

Our basis pathlength recovery method is based on representing the sinogram-to-transmission mapping $g_{i}\left(t_{1},t_{2}\right)$ for DECT as a system of implicit equations where the intersection of the corresponding level curves gives the $t_{1}$ and $t_{2}$ that must be recovered. The number of intersections can be determined using the difference of the level curves at the bounds of the domain and the difference curve’s stationary point. Solving for the intersection points can then be found using a combination of the bisection method and Newton’s algorithm.

The isoline pathlength recovery method is notable in that it explicitly handles cases of nonunique fluence transmission mappings. Previous methods have inadvertently assumed that fluence data is always unique, leading to no consideration of how to identify or handle cases of multiple solutions. While the existence of nonunique mappings has been considered by looking at the Jacobian determinant of the sinogram-to-transmission mapping, this is the first work to reconstruct material density maps when the nonunique mappings are present.

This work presented cases where noisey measurements cause a large number of fluence transmissions with no pathlength mapping, introducing a bias to material pathlength solutions where a Gaussian noise model is inadequate at representing solution distributions. Material decomposition processing from DECT data causes significant amplification of noise that will degrade resulting image quality (Macovski et al 1983, Warp and Dobbins 2003, Balda et al 2010). For this reason, mitigating noise is of upmost importance and has been investigated using techniques for optimizing x-ray spectra, advanced post-processing algorithms, and statistical modeling (Petrongolo and Zhu 2015, Patel and Marin 2018, Jiang et al 2020, Parakh et al 2021). The material pathlength bias observed in this work is an additional factor that further degrades image quality and should be considered when trying to minimize image noise. Incorporation of a noise regularization term in the forward model has potential to mitigate effects of the pathlength bias.

There are limitations to the proposed pathlength recovery method. The process for determining the number of solutions relies on there not being more than two intersections for a pair of level curves. Based on empirical evidence, curve pairs that have more than two intersections exist but are extremely rare. The properties of the level curves need to be studied further to better understand their curvature and oscillations. Additionally, once a set of nonunique pathlength solutions are recovered there is not a robust method in place to determine which pathlength combination should be used. For this work solutions were selected according to an arbitrary forced choice which is highly unlikely to always recover the correct pathlength. A robust, adaptive method for selecting the correct pathlength should be considered for future implementations of the method to maximize accuracy.

In conclusion, we develop an isoline pathlength recovery method capable of resolving nonunique mappings of fluence tranmission to material pathlengths in DECT material decomposition. Our method allowed for the demonstration of how nonunique mappings can degrade the accuracy of reconstructed material basis maps and VMI. Our work contributes to the broader understanding of material decomposition.

## Figures and Tables

**Table 1.** Iodine masses and average column concentrations in each contrast region over 100 noise realizations.

Contrast Region	R1	R2	R3	R4

Mass (×10^−5^ mg)				

True Mass	4.3	12.7	21.2	84.9
Est. Mass w/ BG	12.7 ± 0.1	27.7 ± 0.2	36.8 ± 0.3	109 ± 1 (200 ± 1)
Est. Mass w/o BG	4.5 ± 0.1	13.7 ± 0.2	22.7 ± 0.3	95.4 ± 0.3 (187 ± 1)

Column Concentration (mg cm^−2^)				

True Conc.	1.70	5.09	8.49	33.96
Est. Conc. w/ BG	7.39 ± 0.02	11.09 ± 0.03	14.70 ± 0.03	43.75 ± 0.04 (80.32 ± 0.04)
Est. Conc. w/o BG	1.79 ± 0.02	5.50 ± 0.03	9.11 ± 0.03	38.15 ± 0.04 (74.70 ± 0.04)

Values are reported in units of mg and mg cm^−2^ . The iodine solution concentration in each region is noted at the top of each table column. Standard deviation of the mean values are reported. For regions where nonunique fluence values occur, values calculated using the incorrect recovered pathlength are shown in parantheses. (Appreviations: Est. = Estimated, BG = Background, Conc. = Concentration).

**Figure A1.**
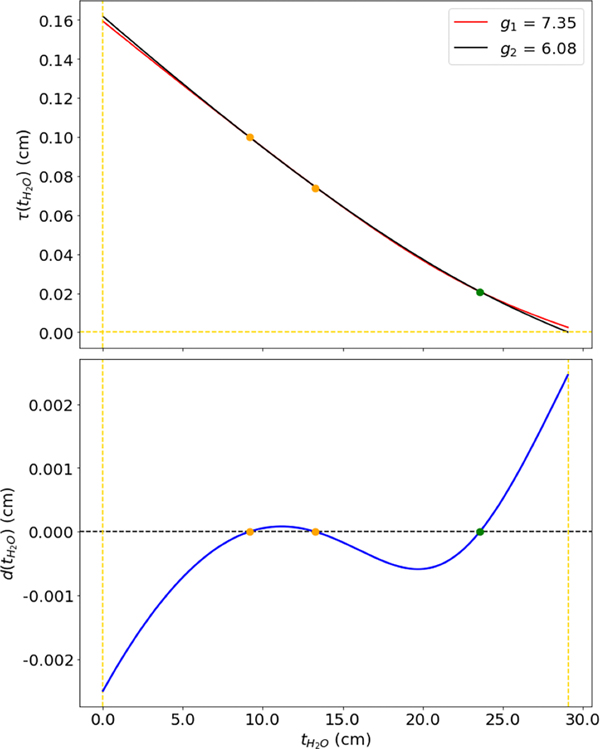
Isoline plots for negative log fluence measurements of water and pure gadolinium using 70 and 120 kV spectra (top). The intersection of the isolines are marked with points indicating solutions of $t_{H_{2}O}=9.16\;cm$ and $t_{G}=0.1\;cm,$ $t_{H_{2}O}=13.28\;cm$ and $t_{G}=0.074\;cm$, and $t_{H_{2}O}=23.58\;cm$ and $t_{G}=2.08\times 10^{-2}\;cm$. The vertical difference of the isolines (bottom) is shown with a green point indicating the solution recovered with the proposed isoline recovery method.

## Data Availability

All data that support the findings of this study are included within the article (and any supplementary information files). Any further distribution of this work must maintain attribution to the author(s) and the title of the work, journal citation and DOI.

## Footnotes

1 Only allowing nonnegative pathlengths introduces a bias when nearly all attenuation is due to only one material. When noise is introduced, recovered pathlength solutions for the scarce material should be distributed around zero. Only allowing positive pathlengths will remove the solutions which correspond to negative pathlength values, causing a bias in the solution distribution.

2 $t_{1}$ and $t_{2}$ are interchangeable here. Since one must be chosen as the variable in $\tau_{i}$, we chose to parameterize $t_{2}$ as a function of $t_{1}$.

## Appendix A. Assumption of solution number

A key assumption made that enables the proposed pathlength recovery method to resolve nonunique transmission mappings is that a set of fluence measurements will map to two pathlength combinations at most based on empirical observation and knowledge that the attenuation forward model is nonlinear. However, this assumption is not always valid due to an additional oscillations of the level curve due to k-edge effects and spectral overlap (Alvarez 2019). It can be shown that the level curves in equation (3) are convex, but slight oscillations of the curves still allow for the two level curves to intersect more than two times and indicates more than two mappings. An example case is shown in figure A1 using water and pure gadolinium where there are three intersections of the level curves. Applying the isoline pathlength recovery method results in one solution being identified at $t_{H_{2}O}=23.58\;cm$ and $t_{G}=2.08\times 10^{-2}\;cm$. For cases of more than two possible pathlength combinations the proposed method fails to find the complete solution set. As such, the capabilities of the isoline pathlength recovery method can be summarized as providing the full solution set for cases of one or two intersections and a partial solution set when more than two roots exist. While generalization to more than two roots is doable, additional checks are needed for every pathlength recovery that will hamper the overall efficiency when completing material decomposition for a full DECT dataset.

## Appendix B. The isoline recovery method algorithm

The full isoline pathlength recovery algorithm depends on a combined use of two root finding methods: bisection and Newton’s method. These two algorithms are utilized regardless of solution number and will appear multiple times in the full pseudocode. As such, we define a high-level function for both bisection and Newton’s method and present the pseudocodes in Algorithm 3 and Algorithm 4 respectively.

**Algorithm 3.** Bisection algorithm for finding the root of a one-dimensional function f(x) over an interval [a,b] for n iterations.

1:	**Function:** Bisection(a,b,f(x),n)
2:	**for** i←1 to n **do**
3:	c←(a+b)/2
4:	**if** f(a)×f(c)<0 **then**
5:	b←c
6:	**else**
7:	a←c
8:	**Return:** c

**Algorithm 4.** Newton’s method algorithm for finding the root of a one-dimensional function f(x) over an interval [a,b] for m iterations.

1:	**Function:** $Newton\left(x_{0},f(x),m\right)$
2:	$x_{i}\leftarrow x_{0}$
3:	**for** i←1 to m **do**
4:	$x_{i+1}\leftarrow x_{i}-f\left(x_{i}\right)/f^{\prime }\left(x_{i}\right)$
5:	$x*\leftarrow x_{m}$
6:	**Return:** x*

To develop the full pathlength recovery algorithm, first we will derive an iterative method for finding the single solution to equation (8). Newton’s method for root finding can again be implemented here. Writing the update step from equation (4) in terms of the implicit functions gives

(B.1)
$$t_{1,n+1}=t_{1,n}-\frac{d\left(t_{1,n}\right)}{d^{\prime }\left(t_{1,n}\right)},$$

where $d^{\prime }\left(t_{1,n}\right)={\left[\frac{dt_{2}}{dt_{1}}\right]}_{1}-{\left[\frac{dt_{2}}{dt_{1}}\right]}_{2}$. Here $[{\frac{dt_{2}}{dt_{1}}]}_{i}$ is equivalent to the difference of the implicit derivatives of $t_{2}$ with respect to $t_{1}$ for $C_{i}\left(t_{1},t_{2}\right)$. To compute the implicit derivative of a two-dimensional equation of the form h(x,y)=0, both sides of the equation must be differentiated with respect to x by applying the chain rule before solving for $\frac{dy}{dx}$:

(B.2)
$$\frac{d}{dx}h(x,y)=\frac{\partial h}{\partial x}+\frac{\partial h}{\partial y}\cdot \frac{dy}{dx}=0$$

(B.3)
$$\frac{dy}{dx}=-\frac{\partial h/\partial x}{\partial h/\partial y}.$$

Applying this formula to equation (3), the derivative ${\left[\frac{dt_{2}}{dt_{1}}\right]}_{i}$ can be composed from the partial derivatives of $C_{i}\left(t_{1},t_{2}\right)$:

(B.4)
$${\left[\frac{dt_{2}}{dt_{1}}\right]}_{i}=-\frac{ℐ\left[\mu_{1},S_{i}\right]}{ℐ\left[\mu_{2},S_{i}\right]},$$

where i=1,2 corresponds to each material. Here we use the operator $ℐ\left[\mu_{j},S_{i};t_{1},t_{2}\right]$ defined in equation (7) but omit the $t_{1}$ and $t_{2}$ arguments for clarity. Inserting into equation (B.1) gives the complete Newton update step for $d\left(t_{1}\right)$.

One issue with using Newton's method is that derivatives of equation (8) are not always monotonic over the entire $t_{1}$ domain, so Newton's method may fail to converge to the single root. An alternative would be to use the bisection method from interval $\left[0,t_{1}^{max}\right]$, but the number of iterations needed to reach floating point accuracy would be inefficient. Since the derivative tends to be well behaved in the neighborhood of root, a combination of bisection and Newton iterations can achieve sufficient accuracy and efficiency. In practice ten bisection and five Newton iterations are adequate.

Next, the location of the stationary point $t_{1}^{sp}$ can be found by using Newton's method. Stationary points are located at the roots of a functions first derivative $f^{\prime }:R\to R$. Newton's method can be used to find the root x*∈R such that $f^{\prime }\left(x*\right)=0$ assuming the existence of a non-vanishing second derivative $f^{\prime \prime }\left(x_{n}\right)\neq 0$ in the area of the root. The iterative update step for Newton's method is

(B.5)
$$x_{n+1}=x_{n}-\frac{f^{\prime }\left(x_{n}\right)}{f^{\prime \prime }\left(x_{n}\right)}.$$

Letting $f^{\prime }\left(x_{n}\right)=d^{\prime }\left(t_{1,n}\right)$ and $f^{\prime \prime }\left(x_{n}\right)=d^{\prime \prime }\left(t_{1,n}\right)$, we have

(B.6)
$$t_{1,n+1}=t_{1,n}-\frac{d^{\prime }\left(t_{1,n}\right)}{d^{\prime \prime }\left(t_{1,n}\right)}.$$

Similar to before, $d^{\prime \prime }\left(t_{1,n}\right)={\left[\frac{d^{2}t_{2}}{dt_{1}^{2}}\right]}_{1}-{\left[\frac{d^{2}t_{2}}{dt_{1}^{2}}\right]}_{2}$. The implicit second derivative of the two-dimensional equation h(x,y)=0 can be found by differentiating $\frac{dy}{\;dx}$ with respect to x using the quotient rule:

(B.7)
$$\frac{d^{2}y}{dx^{2}}=-\frac{\frac{d}{\;dx}\left(\frac{\partial h}{\partial x}\right)\cdot \frac{\partial h}{\partial y}-\frac{\partial h}{\partial x}\cdot \frac{\;d}{\;dx}\left(\frac{\partial h}{\partial y}\right)}{{\left(\frac{\partial h}{\partial y}\right)}^{2}}.$$

The total derivatives of the partial derivatives can be found using the chain rule:

(B.8)
$$\frac{d}{dx}\left(\frac{\partial h}{\partial x}\right)=\frac{\partial^{2}h}{\partial x^{2}}+\frac{\partial^{2}h}{\partial x\partial y}\cdot \frac{\;dy}{\;dx}$$

(B.9)
$$\frac{\;d}{dx}\left(\frac{\partial h}{\partial y}\right)=\frac{\partial^{2}h}{\partial y\partial x}+\frac{\partial^{2}h}{\partial y^{2}}\cdot \frac{\;dy}{\;dx}.$$

Substituting these expressions into the quotient rule expression and simplifying gives

(B.10)
$$\frac{d^{2}y}{dx^{2}}=-\frac{\left(\frac{\partial^{2}h}{\partial x^{2}}+\frac{\partial^{2}h}{\partial x\partial y}\cdot \frac{\;dy}{\;dx}\right)\cdot \frac{\partial h}{\partial y}-\frac{\partial h}{\partial x}\cdot \left(\frac{\partial^{2}h}{\partial y\partial x}+\frac{\partial^{2}h}{\partial y^{2}}\cdot \frac{\;dy}{\;dx}\right)}{{\left(\frac{\partial h}{\partial y}\right)}^{2}}$$

(B.11)
$$=-\frac{\frac{\partial^{2}h}{\partial x^{2}}\cdot \frac{\partial h}{\partial y}+\frac{\partial^{2}h}{\partial x\partial y}\cdot \frac{\;dy}{\;dx}\cdot \frac{\partial h}{\partial y}-\frac{\partial h}{\partial x}\cdot \frac{\partial^{2}h}{\partial y\partial x}-\frac{\partial h}{\partial x}\cdot \frac{\partial^{2}h}{\partial y^{2}}\cdot \frac{\;dy}{\;dx}}{{\left(\frac{\partial h}{\partial y}\right)}^{2}}$$

Finally substituting $\frac{dy}{dx}=-\frac{\partial h/\partial x}{\partial h/\partial y}$ into the expression and simplifying gives

(B.12)
$$\frac{d^{2}y}{dx^{2}}=-\frac{\frac{\partial^{2}h}{\partial x^{2}}{\left(\frac{\partial h}{\partial y}\right)}^{2}-2\frac{\partial^{2}h}{\partial x\partial y}\frac{\partial h}{\partial x}\frac{\partial h}{\partial y}+\frac{\partial^{2}h}{\partial y^{2}}{\left(\frac{\partial h}{\partial x}\right)}^{2}}{{\left(\frac{\partial h}{\partial y}\right)}^{3}}.$$

Applying this formula to equation (3) gives the second derivative:

(B.13)
$${\left[\frac{d^{2}t_{2}}{dt_{1}^{2}}\right]}_{i}=-\frac{ℐ{\left[\mu_{2},S_{i}\right]}^{2}ℐ\left[\mu_{1}^{2},S_{i}\right]-2ℐ\left[\mu_{1},S_{i}\right]ℐ\left[\mu_{2},S_{i}\right]ℐ\left[\mu_{1}\mu_{2},S_{i}\right]+ℐ{\left[\mu_{1},S_{i}\right]}^{2}ℐ\left[\mu_{2}^{2},S_{i}\right]}{ℐ{\left[\mu_{2},S_{i}\right]}^{3}},$$

where k=1,2 but j≠k. Inserting equation (B.13) into equation (B.6) gives the complete Newton update step to find $t_{1}^{sp}$. Due to the derivatives of equation (8) not always being monotonic, strictly using Newton iterations can fail to converge to $t_{1}^{sp}$ just like with the root finding case. The combination of bisection and Newton steps are again needed to converge with sufficient accuracy and efficiency, which is obtainable with ten and five iterations respectively.

The value of $d\left(t_{1}^{sp}\right)$ can now be used to determine if there are zero or two solutions. To solve for both solutions when two exist, we can separate the domain into two intervals that each contain one solution. Using $t_{1}^{sp}$, the $t_{1}$ domain can be divided into the intervals $\left[t_{1}^{min},t_{1}^{sp}\right]$ and $\left[t_{1}^{sp},t_{1}^{max}\right]$ that will each contain one solution. The solution on each interval can then be solved for by using the combination of bisection and Newton's method with ten and five iterations respectively achieving sufficient accuracy.

In the case of no solutions existing, some pathlength combination should still be returned. For these instances, the returned solution pair will be $t_{1}^{sp}$ and the average of $t_{2}$ for each level curve at $t_{1}^{sp}$. Any pathlength combination returned will be inaccurate, but this method returns a solution while requiring minimal additional computation.

## Appendix C. Table of values

**Table C1.** Mathematical notation and definitions used throughout this work.

Symbol	Definition	Units
$V_{1},$ $V_{2}$	Low/high energy potentials for x-ray source tube	kV
$t_{1},$ $t_{2}$	Basis material pathlengths along x-ray path	cm
$\mu_{1}(E),$ $\mu_{2}(E)$	Linear attenuation coefficients	Cm^−1^
$E_{\text{max}}$	Maximum x-ray photon/spectrum energy	keV
$S_{1}(E),$ $S_{2}(E)$	Normalized x-ray energy spectra for $V_{1},V_{2}$	-
$\phi_{i}\left(t_{1},t_{2}\right)$	Relative fluence for spectrum i through ($t_{1},t_{2}$)	-
$g_{i}\left(t_{1},t_{2}\right)$	Sinogram-to-transmission mapping: $-ln\left(\phi_{i}\left(t_{1},t_{2}\right)\right)$	-
$g_{i}^{M}$	Measured sinogram value for spectrum i	-
$C_{i}\left(t_{1},t_{2}\right)$	Isoline equation: $g_{i}\left(t_{1},t_{2}\right)-g_{i}^{M}=0$	-
$\tau_{i}\left(t_{1}\right)$	Implicit function for $t_{2}$ dependent $t_{1}$ for $C_{i}$	cm
$d\left(t_{1}\right)$	Difference of isolines: $\tau_{1}\left(t_{1}\right)-\tau_{2}\left(t_{1}\right)$	cm
$t_{1i}^{min},$ $t_{1i}^{max}$	Minimum/maximum $t_{1}$ for curve i	cm
$t_{2i}^{min},$ $t_{2i}^{max}$	Minimum/maximum $t_{2}$ for curve i	cm
${𝒟}_{i}$	($t_{1},t_{2}$) domain for curve $C_{i}$	-
${𝒟}_{d}$	Domain for $d\left(t_{1}\right)$	-
$t_{1}^{\text{min}},$ $t_{1}^{\text{max}}$	Lower/upper bounds for $t_{1}$ in root search	cm
$t_{1}^{*},$ $t_{1}^{L},$ $t_{1}^{R}$	Solution(s) for $t_{1}$ where $d\left(t_{1}\right)=0$	cm
$t_{1}^{sp}$	Stationary point of $d\left(t_{1}\right)\left(d^{\prime }\left(t_{1}\right)=0\right)$	cm
ℐ[⋅]	Notational operator for fluence model	-
$s_{1},$ $s_{2}$	Basis material sinograms for material 1 and 2	cm
$s_{\text{mono}}$	Monochromatic sinogram	cm
𝒜	Cross-sectional area of pixel	${cm}^{2}$
$t_{ℐ}$	Iodine pathlength in pixel	cm
${𝒱}_{ℐ}$	Iodine volume in pixel	${cm}^{3}$
${ℳ}_{ℐ}$	Iodine mass in pixel	mg
n, m	Iteration number for algorithm steps	-
